# Methods for living evidence synthesis: a systematic review protocol

**DOI:** 10.12688/openreseurope.14044.1

**Published:** 2021-09-28

**Authors:** Ariadna Auladell-Rispau, Josefina Bendersky, Angie Santafe, Cecilia Buchanan, David Rigau Comas, Francisca Verdugo, Camila Ávila, Pablo Alonso, Gerard Urrutia, Gabriel Rada, María Ximena Rojas-Reyes

**Affiliations:** 1 Institut de Recerca de l’ Hospital de la Santa Creu i Sant Pau, Biomedical Research Institute Sant Pau (IIB Sant Pau), Carrer de Sant Quintí, 77, 08041 Barcelona, Spain; 2 Master Program in Clinical Research Applied to Health Science, School of Medicine, Universitat Autònoma de Barcelona, Carrer de Sant Antoni Maria Claret, 167, 08025 Barcelona, Spain; 3 Doctoral Program on Health Research Methodology, School of Medicine, Universitat Autònoma de Barcelona, Carrer de Sant Antoni Maria Claret, 167, 08025 Barcelona, Spain; 4 Iberoamerican Cochrane Centre, C/ Sant Antoni Maria Claret, 167, Pavelló 18, Planta 0, 08025 Barcelona, Spain; 5 Epistemonikos Foundation, Chile Faculty of Medicine, Av. Holanda 895, Providencia, Santiago, Chile; 6 CIBER de Epidemiología Clínica y Salud Pública (CIBERESP), Av. Monforte de Lemos, 3-5, Pabellón 11, Planta 0 28029, Madrid, Spain; 7 Pontificia Universidad Católica de Chile, Av Libertador Bernardo O’ Higgins 340, Santiago, Región Metropolitana, Chile

**Keywords:** Systematic review, Living Systematic Review, Evidence-based medicine, Evidence Synthesis, Living Evidence Synthesis, Living network metanalysis

## Abstract

**Background**: Living evidence (LE) refers to the methodological process that permits new research findings to be continually incorporated to evidence synthesis as they become available. This approach is of great value in the resolution of relevant and rapidly changing clinical questions. To date, the methods to carry out this type of synthesis are not completely defined, and great variability is observed in the approaches used by different groups of authors.

**Objective:** To identify and summarise the current methods used for living evidence synthesis.

**Methods: **We will conduct a systematic literature review of systematic reviews, overviews, and network metanalyses that have used “living evidence synthesis” as part of their methods. The search will be conducted in Medline (via PubMed) and the Epistemonikos database. Two reviewers will independently screen each article for eligibility, extract data, and assess the methodological quality standards of the study accordingly. This protocol is being registered in Prospero.

## Plain language summary

“Living Evidence” refers to a new method for developing synthesis of research evidence, that allows to maintain the information up to date, as new evidence is constantly incorporating as soon as it appears. The basis for the “Living Evidence” has been established, however, the way it is carried out and the methods used by investigators are still unknown.

The objective of this review is to assess and summarise the methods and procedures used by authors to keep the evidence “living”.

In order to meet our objective, we will search for all types of articles reporting “Living Evidence” synthesis to answer a health question. We will search for them in different databases and select the studies that meet the characteristics for “Living Evidence” established in advance. We will extract the information related to the methods used by authors to constantly identify new evidence, and determine how to incorporate the new findings in previous evidence synthesis. This information will be analysed and presented in a descriptive way.

## Introduction

The exponential proliferation of scientific studies and their dispersion through a multitude of scientific journals poses a significant challenge to professionals, given their limited time to keep permanently updated in their respective disciplines. Therefore, systematic reviews (SR) and other derived evidence synthesis products (e.g., clinical practice guidelines [CPG]) are valuable informational tools that try to bring scientific evidence closer to the end user, facilitating its interpretation and application ^1^. However, SRs often do not have the expected impact ^2– 5^. Among various reasons that could be mentioned, the rapid outdating of their conclusions ^6^ is the one that most limits its use and potential impact, making the enormous efforts of developing them largely sterile.

There are areas of high scientific productivity, due to the public health importance of the condition, or the high level of development and technological innovation dedicated to it. It is in these areas where using outdated information poses a major challenge to clinical practice, and solutions are urgently needed.

The methodological approach known as “living evidence” (LE) has emerged as a tool with the potential to address this need. It has been applied mainly to SRs, but currently it is not uncommon to find other types of evidence synthesis such as overviews or network meta-analysis presented as "living".

 A “living systematic review” is a SR that is continually updated, incorporating new relevant evidence as it becomes available. In practice, this means a continual surveillance for new research evidence through ongoing or frequent searches, and the inclusion of new information into the review in a timely manner so that the findings and conclusions of the SR remain current ^7^. When the LE approach is applied to the resolution of relevant and rapidly changing clinical questions, it ensures a rapid update of evidence synthesis that informs on the effects of controversial health interventions, and/or where there are uncertainties ^8^.

The potential for LE to reduce the time and cost of updating evidence-based products and making it available to inform practice is enormous; nevertheless, it is necessary to weigh the potential benefits with the overload of time and resources that living synthesis entails.

The foundations of the LE model for SRs were initially stated in 2014 ^7^ and have been under development over the past few years ^9– 11^. These have been used as the basis for the development and application of new strategies to keep the evidence supporting different products up to date. It has been of particular interest for the synthesis and dissemination of the exponential research output publishing during the COVID-19 pandemic ^12^. However, little guidance exists about the methods to be followed, and their validity and efficiency in achieving the appropriate integration of the studies that emerge every day in the already existing synthesis of evidence. There is also no clear route regarding the editorial processes that the periodic updates of the LE synthesis must follow.

With this SR we seek to identify and summarise the methods and procedures that different groups are currently using to generate and keep the evidence “living” and, to identify those aspects that permits assess the reliability and maintenance of the methodological quality of the synthesis through its multiple updates. We also aim to identify the different actions that current authors of living evidence synthesis are being carried out to alert readers of the evidence changes or periodic updates of the evidence synthesis, particularly of those that may have implications on clinical decisions, including the editorial processes, the informal alerts through evidence-based repositories, among others.

This systematic review is conducted as part of a larger knowledge transfer and a capacity- building project entitled “Living Evidence to Inform Health Decisions” ^13^, funded by the European Union’s Horizon 2020 research and innovation Marie Skłodowska-Curie programme (grant agreement No 894990). Results of this systematic review will contribute to the development of a LE implementation framework for health system organizations to use and incorporate the LE synthesis in the development of knowledge transfer products.

## Protocol

This review complies with the ‘Preferred Reporting Items for Systematic reviews and Meta-Analyses’ (PRISMA) guidelines ^14^ and will be carried out following recommendations outlined in The Cochrane Handbook of Systematic Review of Interventions ^15^.

### Search strategies

***Electronic searches***. Our main search source will be the Epistemonikos database, a comprehensive database of systematic reviews and other types of evidence, maintained by screening multiple information sources to identify SRs and their included primary studies, including the Cochrane Database of Systematic Reviews, Pubmed/MEDLINE, EMBASE, CINAHL, PsycINFO, LILACS, DARE, HTA Database, Campbell database, JBI Database of Systematic Reviews and Implementation Reports, EPPI-Centre Evidence Library ^16^. An additional search using a highly sensitive search strategy will be performed on PubMed/MEDLINE, in order to compare results and be able to identify additional evidence synthesis reports not obtained from searches in Epistemonikos.

The searches will cover from the inception date of each database. No publication status or language restriction will be applied to the searches in Epistemonikos or to the additional searches.

The boolean search strategy that will be used and adjusted to each database is presented in Table 1.

**Table 1.** Search strategy.

**1**	(continuous updat*[tiab] OR continually updat*[tiab] OR constant updat*[tiab]) AND (systematic[sb] OR review[tiab] OR meta analys*[tiab] OR evidence[tiab] OR synthes*[tiab])
**2**	((living systematic review*[tiab]) OR (living meta analys*[tiab]) OR (living rapid review*[tiab]) OR (living rapid evidence[tiab]) OR (living review[tiab]) OR (living network[tiab]) OR (living evidence[tiab]) OR ((continuous updat*[tiab] OR continually updat*[tiab] OR constant updat*[tiab]))
**3**	((systematic[sb] OR review[tiab] OR meta analys*[tiab] OR evidence[tiab] OR synthes*[tiab] )OR (synthes* OR review* OR network* OR evidenc* OR "systematic-review" OR "meta-analysis" OR "meta-analysis" OR "meta-analyses" OR metaanalys* OR "rapid-review" OR "rapid-evidence" OR overview* OR scoping*))

We will update the searches in Epistemonikos prior to the publication of this review, looking for updates to the already identified living evidence synthesis.

***Additional searches***. In order to identify articles that might have been missed in the electronic searches, we will email the contact authors of all the included studies to ask for additional publications of their SRs updates, particularly when there is no update published within a year from the last publication. 

### Eligibility criteria

***Types of studies***. We will include all types of LE synthesis reports. Any of the study types corresponding to the terms “Systematic review”; “meta-analysis”; “rapid review”; “review”; “network”; “network meta-analysis”, “scoping review”, “overview” and “evidence synthesis” will be included if it is linked to any of the following words or descriptions regarding the use of living strategy: “continuous updating”, "continuously updated", "constantly updated", "continual updating", "continually updated", "regular updating", "regularly updated", "updated regularly", "regular updates", "frequent updating", "frequently updated", "periodically updated", "updated periodically", "updated annually", "annually updated", “living”.

Each systematic review/overview and other study types previously mentioned, and subsequent publications of the same review, arising because of the continuous "living" update process, will be included as a single study. However, each one of the subsequent publications of the same study will be reviewed in search of changes in the applied methodology, as well as to obtain information on the editorial implications

The selected articles must describe the methods used to keep the SR constantly updated. The method may either be completely new or may offer a better version of an existing method. The method does not need to have been well tested or used in a way that proves its value.

As a methodological review, our “intervention of interest” is the use of living evidence synthesis as part of the methods of a review assessing the evidence used for answering a clinical question, and therefore, we will exclude methodological or guidance papers.

***Types of participants, interventions, and outcomes***. Because our aim is to review the methodological approach used by authors to produce and maintain living evidence synthesis, we will include the studies (see "type of studies to be included") addressing any type of condition and regardless of the participants or populations they have studied, the interventions or the exposures they have assessed, and the alternatives against which the intervention/exposure are being compared.

### Selection of studies

We will use the L.OVE platform ^17^ to manage the results from the search on Epistemonikos, and Covidence ^18^ to manage the results from the search on MEDLINE (via Pubmed). In both, the duplicate references will be identified and eliminated by an automated process. A special algorithm comparing unique identifiers (database ID, DOI), and citation details (i.e., author names, journal, year of publication, volume, number, pages, article title and article abstract) will be implemented to ensure updates of the same study will not be identified as duplicates of the original publication. We will compare the final results of both processes as a validation of the sensitivity of the search strategy to identify "living evidence synthesis" as well as the sources of consultation (i.e. MEDLINE and Epistemonikos). The results will then be merged into a single list of potentially eligible studies.

Two researchers (AA and JB) will independently screen the search results based on the title and abstract and confirm eligibility based on the inclusion criteria. We will retrieve the full-text article of the references that seems to meet the eligibility criteria or that require further analysis, to decide on their inclusion. Disagreements will be solved by reaching a consensus between reviewers. Each of the included articles and subsequent publications of the same review, arising as a result of the continuous "living" update process, will be linked and included as a single study. As part of the process of linking all publications of the same study, we will identify the original publication, the subsequent publications, and search manually for unpublished updates. In the case that the original publication was not identified as part of our search (e.g., was not originally published as living or to be continuously updated), we will run a special search to identify whether it is using related references, authors names, or title words; if necessary, we will perform a manual search.

We will report the results of this process in the PRISMA flowchart, where we will specify the reasons for any exclusion of the studies obtained in full text. We will include a modification of the PRISMA flowchart to be able to show not only the included articles but also their subsequent updating publications.

### Data extraction

Two reviewers will independently extract data from each included article using a standardized form. We will extract data of the applied methodology for each of the articles relating to the same study (i.e. from all identified publications relating to the same study). We will search for changes in the initially proposed methodology or changes in methodological quality through the updates. The variables to be extracted have been defined by the present review’s authors, based on previous knowledge and developments for LE synthesis identified as part of the methodological literature review ^9– 12, 19, 20^ (see Table 2). Additional data will be extracted to allow the evaluation of the methodological aspects throughout the set of publications of the same study (e.g. the first publication of the synthesis and its subsequent updates) [see Table 3]. Extracted data will be presented in tables with key explanatory information.

**Table 2.** Data to be extracted from each study.

Variable	Description/operational definition
**Study ID**	[Author Year] Study identification for each study
**Title**	Title of the article
**Publication year**	Year of the current article
**DOI**	DOI of this article
**Publication**	[1:Original/2:Update] To write 1 if this article is the first publication of the study; To write 2 if this article is an update
**Identified through eligibility criteria**	[Yes/No] To write Yes if this publication was identified through eligibility criteria; To write No if this publication was identified through additional manual search
**Study design**	[SR/LSR/Living Overview/LNMA] Study design of the article
**Research group**	[Cochrane/ other research group [define]/not reported] Research group involved in the article
**Journal/Editorial Group/Publisher**	Journal where the article has been published
**Definition living methodology**	[author of the reference/other(define)/NR] Definition used to describe living methodology if any
**No. of authors maintaining the ** **living process**	[no. authors/NR] Number of authors involved in the living evidence synthesis
**No. of databases used**	Number and name of databases used during the search
**Grey literature search**	[Yes/No] To write Yes if they specify a grey literature search; to write No if do not specify a grey literature search
**Who has performed the searches**	[authors/specialized technician/external[contracted]/not reported] Person responsible to run the search
**Technological enablers supporting** ** searches**	[yes/no/NR] Specify with yes or no if they used any technological enable to support the search
**If yes, describe**	Describe what technological enablers used to support the search
**Search frequency**	The frequency with which evidence searches are carried out (surveillance process)
**Frequency of the updates**	Frequency with which updates are planned (i.e. incorporate new data to the evidence synthesis)
**Technological enablers supporting** ** surveillance**	[yes/no/NR] Specify if they use technological enablers that support the continue surveillance of the literature
**If yes, describe**	Describe which technological enablers that support the continue surveillance of the literature used
**Funding**	[define/none] Description of funding used to do the living evidence synthesis

**Table 3.** Variables to be extracted across publications of same study/review.

Variable	Description
**Study ID**	Study identification (number) for the group of publications
**Number of updates**	Number of updates from the first publication
**Number of updated publications**	Number of articles published from the first publication
**Information publication agreement** ** available?**	[Yes/no] To write Yes if there is information about an information publication agreement available; To write No if there is no information about an information publication agreement available
**Is there any change on the databases** ** used during updates?**	[yes/no] To write Yes if there is any change on the databases used during updates; to write No if there is no changes on the databases used during updates
**If yes, which changes?**	[define/NA] Describe which changes if any
**Is there any change on the search ** **strategy used during updates?**	[yes/no] To write Yes if there is any change on the search strategy used during updates; to write No if there is no changes on the search strategy used during updates
**If yes, which changes?**	[define/NA] Describe which changes if any
**Is there any other type of change on the ** **methodology section during updates?**	[Yes/No] To write Yes if there is any other change on the methodology section used during updates; to write No if there is not any other change on the methodology section used during updates
**If yes, which changes**	[define/NA] To define any other change found in the methodology section from the first publication to the last update.
**PRISMA updated?**	[Yes/No] To write Yes if the PRISMA flowchart has been updated after each searching/ screening process; to write No if the PRISMA flowchart has not been updated
**Integration of new evidence**	[new republication/web-based/NR] Specify if the new evidence has been integrated through a new republication, an update web-based platform or not reported
**Communicating review status**	[web-based/other (define)/NR] To describe how is communicated to the readers that a new update is available
**Editorial and peer review**	[1: process is completed/2: not done/3: only alert/4: NR] To describe how the editorial and peer review is done for the original and for the updates.

### Assessment of the methodological quality

We will assess the methodological quality of all included studies (e.g. SRs, overviews, or network meta-analysis) using the AMSTAR II checklist ^21^ and other appropriate instruments according to the type of study (e.g. the NCCMT Quality Assessment Tool-Review articles ^22^). The assessment will be carried out on the original publication and all its subsequent updated publications. Two independent authors will complete the assessment of each included study (original and its linked publications); a third author will discuss the final assessment with both authors who performed the initial evaluation and solve disagreements. We will present the results of this assessment in descriptive tables. We will pay particular attention to changes in the methodological quality through publications of the same study.

### Strategy for data synthesis

To present the data's basic features in the study, we will do a descriptive analysis (using frequencies, numbers, and proportions). We will present the results in tables, both for each selected article and for the group of publications linked to the same study. Qualitative data (e.g., description of the living methodology) will be presented in tables.

We will report the results in subgroups according to the type of study (LSR, Living overview, or LNMA).

### PROSPERO registration

This protocol is registered under the accession number CRD42021248963.

### Ethical considerations

As researchers will not access information that could lead to the identification of an individual participant, obtaining ethical approval was waived.

### Data sharing

All data related to the project will be available. Epistemonikos Foundation will grant access to data.

## Conclusions

The current way authors are incorporating LE in the systematic reviews or other type of evidence synthesis is unknown. We are going to conduct a systematic review of studies reporting LE synthesis as part of the methods to solve health questions including systematic reviews, network meta-analysis and living overviews.

The aim of this review is to identify and summarise the methods and procedures that different groups are currently using to generate and keep the evidence “living” and, to identify those aspects that permits assess the reliability and maintenance of the methodological quality of the synthesis through its multiple updates.

## Data availability

### Underlying data

No data are associated with this article.

### Reporting guidelines

Open Science Framework: PRISMA-P checklist for “Methods for living evidence synthesis: a systematic review protocol”. https://doi.org/10.17605/OSF.IO/2QA4U ^23^

Data are available under the terms of the Creative Commons Zero "No rights reserved" data waiver (CC0 1.0 Public domain dedication).
