# Methodological approaches for developing and reporting living evidence synthesis: a study protocol

**DOI:** 10.12688/openreseurope.14044.2

**Published:** 2022-03-21

**Authors:** Ariadna Auladell-Rispau, Josefina Bendersky, Angie Santafe, Cecilia Buchanan, David Rigau Comas, Francisca Verdugo, Camila Ávila, Pablo Alonso, Gerard Urrutia, Gabriel Rada, María Ximena Rojas-Reyes

**Affiliations:** 1 Institut de Recerca de l’ Hospital de la Santa Creu i Sant Pau, Biomedical Research Institute Sant Pau (IIB Sant Pau), Carrer de Sant Quintí, 77, 08041 Barcelona, Spain; 2 Master Program in Clinical Research Applied to Health Science, School of Medicine, Universitat Autònoma de Barcelona, Carrer de Sant Antoni Maria Claret, 167, 08025 Barcelona, Spain; 3 Doctoral Program on Health Research Methodology, School of Medicine, Universitat Autònoma de Barcelona, Carrer de Sant Antoni Maria Claret, 167, 08025 Barcelona, Spain; 4 Iberoamerican Cochrane Centre, C/ Sant Antoni Maria Claret, 167, Pavelló 18, Planta 0, 08025 Barcelona, Spain; 5 Epistemonikos Foundation, Chile Faculty of Medicine, Av. Holanda 895, Providencia, Santiago, Chile; 6 CIBER de Epidemiología Clínica y Salud Pública (CIBERESP), Av. Monforte de Lemos, 3-5, Pabellón 11, Planta 0 28029, Madrid, Spain; 7 Pontificia Universidad Católica de Chile, Av Libertador Bernardo O’ Higgins 340, Santiago, Región Metropolitana, Chile

**Keywords:** Systematic review, Living Systematic Review, Evidence-based medicine, Evidence Synthesis, Living Evidence Synthesis, Living network metanalysis

## Abstract

**Background**: Living evidence (LE) refers to the methodological processes that permit new research findings to be continually incorporated into evidence synthesis. This approach is of great value in the resolution of relevant and rapidly changing clinical questions. To date, the methods to carry out this type of synthesis are not completely defined, and great variability is observed in the approaches used by different groups of authors.

**Objective: **To identify, evaluate and summarise the current methods used for living evidence synthesis

**Methods: **We will conduct a methodological study based on a systematic literature search to identify any type of evidence synthesis such as systematic reviews, network metanalyses and overviews that used “living evidence synthesis” as part of their methods. The search will be conducted in Medline (via PubMed) and Epistemonikos databases. Additionally, we will search websites of the organisations publishing any living evidence synthesis retrieved in the two databases, in order to identify unpublished subsequent reports. Two reviewers will independently assess each article against the selection criteria, extract data on methods and procedures, and assess the methodological quality of each publication. Data will be analysed descriptively.

## Plain language summary

“Living Evidence” refers to a new method for developing synthesis of research evidence that allows maintaining the information up to date, as new evidence is constantly incorporated as soon as it appears. The basis for the “Living Evidence” has been established, however, the way it is carried out and the methods used by investigators are still unknown.

This study aims to identify, evaluate and summarise the methods and procedures used by authors to keep the evidence “living”.

In order to meet our objective, we will search for all articles reporting a living evidence synthesis to answer a health question. We will search for them in different databases and select the reviews that meet “Living Evidence” characteristics established in advance. We will extract the information related to the methods used by authors to constantly identify new evidence, the procedures used to incorporate the new findings in previous evidence synthesis, and the process followed to inform users of updates. This information will be analysed and presented in a descriptively.

## Introduction

The exponential proliferation of scientific studies and their dispersion through a multitude of scientific journals, some of them of limited access for readers, poses a significant challenge to health care professionals, given their limited time to keep permanently updated in their respective disciplines. Therefore, systematic reviews (SR) and other derived evidence synthesis products (e.g., overwies of reviews, network meta-analysis) are valuable informational tools that try to bring scientific evidence closer to the end-user, facilitating its interpretation and application ^1^. However, SRs often do not have the expected impact ^2– 5^; among various reasons that could be mentioned, the rapid outdating of their conclusions is the one that most limits its use and potential impact ^6^, making the enormous efforts of developing them largely sterile.

There are areas of high scientific productivity and controversy in which using outdated information poses a major challenge to clinical practice, and solutions are urgently needed. The methodological approach known as “living evidence” (LE) has emerged as a tool with the potential to address this need. It has been applied mainly to SRs, but currently, it is not uncommon to find other types of evidence synthesis such as overviews and network meta-analysis presented as "living".

A “living systematic review” is a continually updated SR, which incorporates new relevant evidence as it becomes available. In practice, this means continual surveillance for new research evidence through on going or frequent searches, and the inclusion of new information into the review in a timely manner so that the findings and conclusions of the SR remain current ^7^. When the LE approach is applied to the resolution of relevant and rapidly changing clinical questions, it ensures a rapid update of evidence synthesis that informs on the effects of controversial health interventions, and/or where there are uncertainties ^8^.

The potential for LE to reduce the time and cost of updating evidence-based products such as clinical practice guidelines, health technology evaluations and evidence summaries aimed to inform health decisions (e.g. policy briefs) and making it available to inform practice is enormous; nevertheless, it is necessary to weigh the potential benefits with the overload of time and resources that living synthesis entails.

The foundations of the LE model for SRs were initially stated in 2014 ^7^ and have been under development over the past few years ^8– 11^. These have been used as the basis for the development and application of new strategies to keep the evidence supporting different products up-to-date ^12, 13^. It has been of particular interest to synthesise and disseminate the exponential research output published during the COVID-19 pandemic ^14^. Being a highly attractive methodology to be applied for many questions in health, but equally recent, little is known about the methods followed by the authors, their validity and the efficiency to achieve an adequate integration of emerging studies in the existing evidence synthesis. Despite some advances in this regard ^15^, the methods and quality of living systematic reviews and other types of living evidence synthesis have not been empirically evaluated. There is also no clear route regarding the dissemination process, publication or editorial processes that the periodic updates of the living evidence synthesis follow to reach readers and potential users.

With this study, we seek to identify, evaluate, and summarise the methods and procedures that different groups are currently using to generate and keep the evidence synthesis “living”, and identify those aspects associated with the reliability and maintenance of the methodological quality of that synthesis through its multiple updates. We also aim to identify the different actions carrying out by living evidence synthesis authors to alert users of the evidence changes or periodic updates of the evidence synthesis, particularly when they have implications on clinical decisions and health policies. In this regard, we will explore editorial processes for new publications, informal update alerts, use of web-based repositories, among other actions presented by authors. Finally, we aim to provide suggestions for improving the development and report of living evidence synthesis based on a previous work done by our research team, in which we have reviewed all the methodological and guidance papers published up to July 2021 describing the “living evidence methodology” ^7– 11^, in order to identify the relevant methodological items that a living evidence synthesis should include.

This study is being conducted as part of a larger knowledge transfer project entitled “Living Evidence to Inform Health Decisions”, funded by the European Union’s Horizon 2020 research and innovation Marie Skłodowska-Curie Action (grant agreement No. 894990). Results of this assessment will contribute to the development of a living evidence implementation framework for health system organizations to use and incorporate LE methodology in the development of knowledge transfer products.

### Study design

This is a cross sectional methodological study in which we will collect data from all living evidence synthesis published reports (articles) identified in the current available literature.

### Study population

Our population of interest is any type of evidence synthesis including systematic reviews, meta-analysis, rapid review, network metanalysis, and overviews that have reported to use "living evidence" (LE) methodology (i.e. living evidence synthesis reports) to answer any clinical questions regardless the LE methodology used.

Because our aim is to review the methods used by authors to produce and maintain living evidence synthesis, we will include all “living evidence synthesis” identified, regardless of the type of condition, the participants or populations, the interventions or the exposures, and the alternatives against which the interventions/exposures are being compared.

### Selection criteria

Any of the review types corresponding to the terms “systematic review”; “meta-analysis”; “rapid review”; “review”; “network”; “network meta-analysis”, “scoping review”, “overview” and “evidence synthesis” will be selected if it includes any of the following words or descriptions regarding the use of a living strategy: “continuous updating”, “continuously updated”, “constantly updated”, “continual updating”, “continually updated”, “regular updating”, “regularly updated”, “updated regularly”, “regular updates”, “frequent updating”, “frequently updated”, “periodically updated”, “updated periodically”, “updated annually”, “annually updated” or “living”.

Each report meeting the previously mentioned characteristics and the subsequent publications of the same review will be included and treated as a single study (i.e. review /evidence synthesis), considering the initial publication as the baseline one and the subsequent publications as its updates. Nevertheless all review reports will be assessed independently in order to collect the data of interest (see data collection).

### Identification of living evidence synthesis reports

The identification of the evidence synthesis of interest will be carried out through systematic and exhaustive searches in the biomedical databases ^16^. With this aim we will use Epistemonikos database as main source; this is a comprehensive database of systematic reviews and other types of evidence synthesis reports, maintained by screening multiple information sources to identify SRs and their included primary studies, including the Cochrane Database of Systematic Reviews, Pubmed/MEDLINE, EMBASE, CINAHL, PsycINFO, LILACS, DARE, HTA Database, Campbell database, JBI Database of Systematic Reviews and Implementation Reports and EPPI-Centre Evidence Library ^17^. This database has been validated, showing its completeness in collecting the published SRs and other type of evidence syntheses ^18^.

The searches will cover from the inception date. No publication status or language restriction will be applied to the searches in Epistemonikos. The boolean literature search strategy that will be used is presented in Table 1.

**Table 1.** Search strategy.

**1**	(continuous updat*[tiab] OR continually updat*[tiab] OR constant updat*[tiab]) AND (systematic[sb] OR review[tiab] OR meta analys*[tiab] OR evidence[tiab] OR synthes*[tiab])
**2**	((living systematic review*[tiab]) OR (living meta analys*[tiab]) OR (living rapid review*[tiab]) OR (living rapid evidence[tiab]) OR (living review[tiab]) OR (living network[tiab]) OR (living evidence[tiab]) OR ((continuous updat*[tiab] OR continually updat*[tiab] OR constant updat*[tiab]))
**3**	((systematic[sb] OR review[tiab] OR meta analys*[tiab] OR evidence[tiab] OR synthes*[tiab] )OR (synthes* OR review* OR network* OR evidenc* OR "systematic-review" OR "meta-analysis" OR "meta-analysis" OR "meta-analyses" OR metaanalys* OR "rapid-review" OR "rapid-evidence" OR overview* OR scoping*))

We will update the searches in Epistemonikos prior to the publication of this review, looking for updates to the already identified living evidence synthesis.

***Additional searches.*** In order to identify new articles or update reports that might have been missed in the electronic searches, we will manually search websites of the organizations reporting the living evidence synthesis identified in electronic searches and email the contact authors of all the included reviews to ask for publications of their updates, particularly when there is no a published update identified within a year from the last publication. 

### Sampling and selection

Results from these searches will be automatically included in the Living Overview of Evidence (L.OVE) platform ^19^ of the Epistemonikos Foundation. This platform has been validated as a repository for the COVID-19 showing to be a highly comprehensive source of evidence ^20^, where the duplicate references will be identified and eliminated by an automated process. A special algorithm comparing unique identifiers (database ID, DOI) and citation details (i.e., author names, journal, year of publication, volume, number, pages, article title and article abstract) will be implemented to ensure updates of the same review will not be identified as duplicates of the original publication.

Two researchers (AA and JB) will independently screen the search results based on the title and abstract and confirm eligibility according to the selection criteria. We will retrieve the full-text article of the references that meet the eligibility criteria or require further analysis, to decide on their inclusion. Disagreements will be solved by reaching a consensus between reviewers.

As it was previously mentioned, each of the included articles and subsequent publications of the same review, arising from the “living evidence” updating process, will be linked and included as a single study (i.e. review /evidence synthesis). As part of this process we will identify the original publication, the subsequent publications and search manually for unpublished updates. In the case that the original publication was not identified as part of our search (e.g., it was not initially published as “living” evidence synthesis or to be continuously updated), we will run necessary searches to identify whether it is, using related references, authors names, or title words; if necessary, we will perform a manual search and contact the review authors.

### Data collection

Five researchers trained in advance will extract data from all the included review reports; each report will be assessed by two professionals who will extract data independently using a specifically designed standardized form to collect the information of interest. This form has been developed on the basis of previous work done by our research team, that reviewed all methodological and guidance papers published up to July 2021 describing a “living evidence” methodology ^7– 13, 20, 21^ in order to identify the relevant methodological items to be collected. Based on this review, we developed a preliminary list of variables to be collected as part of this study that were later validated by the research group experts in the field.

The final list of variables to be collected (see Table 2) includes variables from each review report and other variables specifically from the baseline report and from its updates. There are other set of variables (see Table 3) to be collected from the same review (e.g. the baseline publication of the review and its subsequent updates) to allow for the evaluation of other “living evidence process” related aspects . The form developed to collect all these data was piloted in a sample of five living evidence synthesis reports.

**Table 2.** Variables to be collected.

Data to be collected from all the reports/articles identified	
Variable	Description/operational definition
**Study ID**	A study identification number will be assigned to the set of publications of the same review, authoring by the same group or research team
**Title**	Title of the article
**Publication year**	Year of the current article
**DOI**	DOI of this article
**Journal/Editorial Group/Publisher**	Journal where the article has been published
**Journal Impact Factor**	The impact factor of journal where the systematic living evidence synthesis are being published
**Funding**	Description of funding supporting the review /evidence synthesis and the evidence surveillance as part of the living evidence approach
**Source of identification**	To define whether this article/report has been identified from electronic searches (databases), registers or other sources (i.e. organizations website or repositories among others).
**Type of evidence synthesis report**	To define whether this article/report is a systematic review, an overview, network metanalysis or other type of evidence synthesis.
**Type of Publication**	To define whether or not it is the original (first) publication of the review/ evidence synthesis or a publication of one of its updates
**Is the question to be answered clearly stated?**	To define whether or not the review authors explicitly state the question and the subgroup of interest
**Are the end points or outcomes of interest** **clearly defined (as part of the protocol or in the** **text of the manuscript)?**	To define whether or not the review authors explicitly state the outcomes of interest, including characteristics of its measure
**Use of a “living evidence” approach**	To define whether or not the review authors explicitly state they are applying or will apply a living evidence approach to solve the question
**Definition used for the living methodology**	To define whether or not the review authors explicitly present a definition to describe the living methodology.
**Reference(s) that supports the methodological** ** approach used**	[author of the reference/other(define)/NR]
**Search strategy reported**	To define whether or not the review authors present a detailed search strategy
**Evidence sources searched (name of data bases ** **used)**	To define whether or not the review authors present the data bases searched/to be searched
**Grey literature search performed**	To define whether or not authors carried out grey literature search
**Searching for ongoing trials in registers**	To define whether or not authors carried out searches in trials registers
**Who is performing the searches?**	To define who is in charge of running searches [authors/specialized technician/ external[contracted]/ Other/NR]
**Are the selection criteria clearly defined?**	To define whether or not the review authors explicitly state the selection criteria for the studies, including the type of study design and publication type
**Included studies reporting on the outcomes of ** **interest**	To define whether or not the review authors identified studies reporting on the outcomes of interest * *Information of the outcomes with no evidence will be collected
**Evidence synthesis for each outcome**	To define whether the review authors conducted a metanalysis or a narrative evidence synthesis for summarising the evidence found for the outcomes of interest
**Methodological quality**	AMSTAR II assessment result
**Rational for setting up/or preforming a living ** **evidence synthesis**	To define whether or not the review authors clearly state the reasons to conduct a living evidence synthesis (setting up or continue the living process) * * *information on the appropriateness of the statements will be collected according* * to predefined criteria*
Data to be collected from the baseline report	
Variable	Description/operational definition
**Planning for evidence monitoring /surveillance**	To define whether or not the review authors defined in advance (in protocol or in the article text) any of the following aspects as part of evidence surveillance or monitoring plan: - Type of studies to be identified as part of evidence surveillance - Frequency of electronic searches during evidence monitoring - Frequency of electronic searches in registers during evidence monitoring - Frequency of screening and selection of the new evidence identified by the searches during evidence monitoring - Type of publications included as part of the during evidence monitoring - Anticipate duration of the evidence monitoring - The statistical methods used for updating the metanalyses when new evidence becomes available- integrating new data-? - Statistical considerations regarding repeated analysis of accumulating primary trial data - Considerations and rules to stop the evidence monitoring for the given question of interest (e.g. improvement of evidence quality for main outcomes; question no longer relevant) - Information of how often the original question is going to be revisited?
**Resources to maintain the living evidence processes**	To tefine whether or not the review authors have defined in advance any of the following aspects as part of the evidence monitoring plan: - number of authors involved in the living evidence synthesis; or whether there is no information provided - Is there any assigned person/role in charge of creation and maintenance the search strategy? - Is there any assigned person/role in charge of performing the evidence searches? - Is there any assigned person/role in charge of screening search results during evidence monitoring - Is there any assigned person/role in charge of performed the Risk of Bias (RoB) assessment task? - Is there any assigned person/role in charge of performed extracting the new study data? - Is there any description of technological enablers supporting searches? * - Is there any description of technological enablers supporting evidence screening?* * Description of enablers used for searches and/or for screening will be collected
Data to be collected from the updates publication or reports	
Variable	Description/operational definition
**Changes in methodology from the baseline report**	Define whether or not the review authors provided information on changes from the baseline or the last report in any of the following: - Evidence sources (i.e. new databases or registers searched) - Selection criteria used for eligible studies - Type of studies to be identified as part of evidence surveillance - Frequency of electronic searches during evidence monitoring - Frequency of electronic searches in registers during evidence monitoring - Frequency of screening and selection of the new evidence identified by the searches during evidence monitoring - Type of publications included as part of the during evidence monitoring
**Information to the continuous update of the** ** PRISMA flowchart**	Define whether or not the review authors provided information on number of evidence surveillance results from the last update, considering the following: - Information of the number of studies identified by searches since last report? - Independent information of the number of peer reviewed articles and preprints identified since last report? - Information of the number of ongoing studies identified in registers since last report? - Information of the number of studies screened since last report? - Number of eligible studies since last report - Number of new studies included in the analysis
**Information about the integration of new** ** evidence (NEI)**	Define whether or not the review authors provided information on the integration of new evidence, including: - Description of the new study(ies) - RoB assessment of new studies - Outcomes reporting (information of outcomes informed by new evidence) - Statistical methods and considerations taking into account for updating metanalyses integrating new studies data

**Table 3.** Variables and other measures across publications of the same review.

Variable	Description
**Number of publications and/** **or update reports identified**	Number of publications (i.e. baseline report and update)
**Scientif publications identified**	Number of peer reviewed articles published from the first publication
**Other type of update reports** ** identified**	Number of updates identified from other sources than scientific publications (e.g. Website)
**Updates communication and ** **publication**	Information on Updates including any of the following: - Dissemination of content through website, repositories or similar - Alerting readers (describe how is communicated to the readers that a new update is available) - Frequency of communicating updates to readers
**Editorial and peer review**	Define whether or not the review authors provided information on the editorial and peer review process for the baseline report and for the updates. Including: - Is there a Citation/DOI is provided for each publication (i.e. Baseline and updates) - Is there a publication agreement with any journal available - Other
**Revisit parameters through** ** the living evidence process**	Define whether or not the review authors provided information regarding the parameter revisited during the evidence surveillance as part of the LE process, such as: - Revisited the PICO of the question - Revisited parameters of searches - Revisited selection criteria - Monitoring stopping rules
**Changes in the quality of the** ** report (AMSTAR II assessment)**	Define whether or not the review reports quality changed from the updates though its updates. - Improve quality - Decrease quality - There are no changes identified in quality of the reports

To be able of identifying the methodological quality of the baseline reviews and any variations through its multiple updates, as part of the data collection we will assess the methodological quality of all included evidence synthesis reports using the appropriate instruments (i.e. AMSTAR II checklist for systematic reviews ^22^ and NCCMT Quality Assessment Tool, for other review articles ^23^). Two independent authors will complete the assessment on the original publication (baseline review) and subsequent updated reports or publications. Disagreements will be solved by consensus. We will pay particular attention to changes in the methodological quality through publications of the same review. Results of this assessment will be presented in descriptive tables.

### Data analysis

To present the data’s basic features in the evidence synthesis reports, we will perform a descriptive analysis (using frequencies, numbers, and proportions). We will present the results in tables, both for each review report and for the group of publications linked to the same review/evidence synthesis. Qualitative data (e.g., description of the living methodology) will be presented in tables.

We will check the methodological issues reported by the authors against those in the list of variables that we previously defined based on the review of methodological articles.

The aspects related to the updating of the reports, scientific publications or other forms used by authors to disseminate the review updates, will be collected and presented as qualitative information in summary tables, since this is one of the methodological aspects still to be defined in living evidence processes.

If feasible, we will report the results in subgroups by the type of synthesis (LSR, Living overview, or LNMA).

***PROSPERO registration.*** This protocol is registered under the accession number CRD42021248963

***Ethical considerations.*** As researchers will not access information that could lead to the identification of an individual participant, obtaining ethical approval was waived.

***Data sharing.*** All data related to the project will be available. Epistemonikos Foundation will grant access to data.

## Conclusions

The way in which authors are currently incorporating the living evidence approach into systematic reviews and other synthesis products is still unknown.

This project will permit to identify, evaluate and summarise the methods and procedures that different groups are using to generate and keep the evidence “living”.

One of the biggest challenges of setting up a “living evidence” process is to rapidly transfer the results of the updates to end-users, such as clinicians and other decisions makers. There are some proposals done in this regard so far but not a clear route to reach this goal. This project will identify the current practice and methods used by authors to disseminate the constant updates of the evidence synthesis as well as those aspects that will permit the living evidence authors to maintain informed their final users through its multiple updates.

This study will provide information on the compliance of the current authors of the living evidence synthesis with the methodological standards proposed so far, which will serve to improve the living evidence synthesis reports, increase their transparency, as well as guide their potential evaluation.

## Data availability

### Underlying data

No data are associated with this article.

### Reporting guidelines

*Extended data*

Open Science Framework: LIVING EVIDENCE TO INFORM HEALTH DECISIONS. Registration DOI 10.17605/OSF.IO/27MEC

This project contains the following extended data:

*Reporting guidelines*

Open Science Framework: PRISMA-P checklist for “Methodological approaches for developing and reporting living evidence synthesis: a study protocol” https://osf.io/fm6ej/

Data are available under the terms of the Creative Commons Zero "No rights reserved" data waiver (CC0 1.0 Public domain dedication).
