# Supplementary material for: Methodological approaches for developing and reporting living evidence synthesis: a study protocol
Source: Open Res Eur. 2022 Mar 21;1:113. Originally published 2021 Sep 28. [Version 2] doi: 10.12688/openreseurope.14044.2 (PMC10446035; doi:10.12688/openreseurope.14044.2)
Supplement: Peer review history

# Peer review: Reviewer response for version 2

**DOI:** 10.21956/openreseurope.15750.r28886

**Published:** 2022-03-28

**Authors:** Juan Erviti

**Affiliations:** 1 Unit of Innovation and Organization, Navarre Health Service, Pamplona, Spain

I recommend approval of the latest version.

Is the study design appropriate for the research question?

Yes

Is the rationale for, and objectives of, the study clearly described?

Yes

Are sufficient details of the methods provided to allow replication by others?

Yes

Are the datasets clearly presented in a useable and accessible format?

Not applicable

Reviewer Expertise:

NA

I confirm that I have read this submission and believe that I have an appropriate level of expertise to confirm that it is of an acceptable scientific standard.

# Peer review: Reviewer response for version 2

**DOI:** 10.21956/openreseurope.15750.r28885

**Published:** 2022-03-24

**Authors:** Nancy Santesso

**Affiliations:** 1 Department of Health Research Methods, Evidence, and Impact, McMaster University, Hamilton, Canada

I have no further comments to make.

Is the study design appropriate for the research question?

No

Is the rationale for, and objectives of, the study clearly described?

No

Are sufficient details of the methods provided to allow replication by others?

No

Are the datasets clearly presented in a useable and accessible format?

Not applicable

Reviewer Expertise:

Systematic reviews and guideline development

I confirm that I have read this submission and believe that I have an appropriate level of expertise to confirm that it is of an acceptable scientific standard.

# Peer review: Reviewer response for version 1

**DOI:** 10.21956/openreseurope.15135.r27711

**Published:** 2021-10-12

**Authors:** Nancy Santesso

**Affiliations:** 1 Department of Health Research Methods, Evidence, and Impact, McMaster University, Hamilton, Canada

Methods for living reviews are evolving and a study to determine the current methods being used by review authors will be important as we move forward, in particular if compared to the variety of methods being proposed today. Below I have included a few suggestions that may improve the paper.

 I believe that this study is not a methodological review. My understanding of a methodological review is a review of studies that assess the effects of a method used in research (sometimes compared to another method), or association of a method with some outcome, and these effects or associations are pooled together across studies if possible (see Cochrane methodological reviews). To me, instead, this paper is a survey of what methods are currently being used. This may explain why on Page 4, the reference to the  ‘intervention of interest’ does not seem to apply.

 If you change this to a survey, the PRISMA checklist/statement would not be used when reporting this paper.

 Since this study is about the methods for living reviews, I think it should be more broad. The information that will be extracted seems to focus mostly on the search methods, but there are other methods that are or could be affected when conducting a living review. I have mentioned a few below but I think the authors should explore the different methods that are currently being discussed in the literature and expand the data they will collect.

 For example, the authors could consider adding how the results of the search and screening are reported as there seems to be some research about different methods of reporting being used. Alternatively, the authors may decide not to gather this data and instead refer to the paper by Kahale 2021.

 There is also some literature about the statistical methods that could be used in living reviews (see Simmonds 2017). The Simmonds paper identifies many important methods that could be used. The authors have not indicated that they will collect data about different methods of statistical analyses and interpretation, but could. In addition, there is some question about the criteria to decide when re-analysis and a subsequent publication should occur when doing a living review (e.g., it could be when results or certainty of evidence change, or simply when there is new evidence regardless of whether the effect or certainty changes). Data about if and how the authors of the reviews report these methodological decisions would likely be important to collect.

 It’s not clear to me why the methodological quality of the reviews are being assessed. How will this information be used? In addition, do the current tools actually address issues relevant to living reviews? Will these tools provide quality information relevant to a living review in particular?

 A minor comment: I understand that different types of reviews will be included, but the paper may be easier to follow if the living 'reviews' are not referred to as ‘studies’.

**References**

 Kahale LA, Elkhoury R, El Mikati I *et al.* Tailored PRISMA 2020 flow diagrams for living systematic reviews: a methodological survey and a proposal [version 2; peer review: 1 approved, 1 approved with reservations]. *F1000Research* 2021, **10**:192 ( https://doi.org/10.12688/f1000research.51723.2)

 Simmonds M, Salanti G, McKenzie J, Elliott J; Living Systematic Review Network. Living systematic reviews: 3. Statistical methods for updating meta-analyses. J Clin Epidemiol. 2017 Nov; 91:38-46.

Is the study design appropriate for the research question?

No

Is the rationale for, and objectives of, the study clearly described?

No

Are sufficient details of the methods provided to allow replication by others?

No

Are the datasets clearly presented in a useable and accessible format?

Not applicable

Reviewer Expertise:

Systematic reviews and guideline development

I confirm that I have read this submission and believe that I have an appropriate level of expertise to confirm that it is of an acceptable scientific standard, however I have significant reservations, as outlined above.

## References

[1] : Tailored PRISMA 2020 flow diagrams for living systematic reviews: a methodological survey and a proposal. *F1000Research* .2021;10: 10.12688/f1000research.51723.2 10.12688/f1000research.51723.2 PMC880490935136567

[2] : Living systematic reviews: 3. Statistical methods for updating meta-analyses. *J Clin Epidemiol* .2017;91: 10.1016/j.jclinepi.2017.08.008 38-46 10.1016/j.jclinepi.2017.08.008 28912004

# Response

**Published:** 2022-02-22

**Authors:** Maria Ximena Rojas Reyes

**Affiliations:** INSTITUT DE RECERCA DE L'HOSPITAL DE LA SANTA CREU I SANT PAU FUNDACION - IR-HSCSP, Spain

Dr. Santesso, we thank your careful reading of the manuscript and your constructive comments. We have taken them into account in order to improve and clarify the manuscript. Please find below a detailed point-by-point response to your comments. (Reviewer comments in italics)

***Comment 1*** *Methodological review vs methodological survey* *I believe that this study is not a methodological review. My understanding of a methodological review is a review of studies that assess the effects of a method used in research (sometimes compared to another method), or association of a method with some outcome, and these effects or associations are pooled together across studies if possible (see Cochrane methodological reviews). To me, instead, this paper is a survey of what methods are currently being used. This may explain why on Page 4, the reference to the ‘intervention of interest’ does not seem to apply. If you change this to a survey, the PRISMA checklist/statement would not be used when reporting this paper.*

**Reply to comment 1** We have reviewed and is no clear nomenclature in biomedical literature for this type of study aimed to assess the methods used by others. Due to the lack of standardization between the terms “methodological survey”, “methodological review” or “meta-epidemiological study”, we decided to use “methodological study” rather than a “methodological review” to be informative and allow for appropriate indexing. A methodological study is defined as any study that describes or analyzes methods (design, conduct, analysis, or reporting) in published (or unpublished) literature. (Mbuagbaw *et al.)* We have introduced changes in our protocol according to this type of study design, which includes changes in the title and the methods section of this manuscript (see lines 136-147).  Ref: Mbuagbaw, Lawrence & Lawson, Daeria & Puljak, Livia & Allison, David & Thabane, Lehana. (2020). A tutorial on methodological studies: The what, when, how and why. BMC Medical Research Methodology. 20. 226. 10.1186/s12874-020-01107-7.

**Comment 2** *Exploring different methods of conducting a living review. Since this study is about the methods for living reviews, I think it should be broader. The information that will be extracted seems to focus mostly on the search methods, but there are other methods that are or could be affected when conducting a living review. I have mentioned a few below but I think the authors should explore the different methods that are currently being discussed in the literature and expand the data they will collect. For example, the authors could consider adding how the results of the search and screening are reported as there seems to be some research about different methods of reporting being used. Alternatively, the authors may decide not to gather this data and instead refer to the paper by Kahale 2021.*

**Reply to comment 2 **Prior to defining the variables to be collected, we conducted a review of methodological and guidance articles published up to June 2021, all the references you have mentioned but Kahale 2021, were included and reviewed, they were already listed previously as part of the protocol references (see references 7 to 14 and 16, 21). The publication by Kahale 2021 was published later of our initial review but is now included as part of references reviewed: Ref Nº13. Kahale L, Elkhoury R, El Mikati I, et al.: Tailored PRISMA 2020 flow diagrams for living systematic reviews: a methodological survey and a proposal. F1000Research. 2021; 10. Based on the previous work done by other authors, we defined the variables to be collected and exceed those related only to the search methods. This list already includes information about how results of the search and screening are reported, as we presented in tables 2 and 3. Nevertheless, taking into account your comments we have reviewed the list of variables and provided a better explanation of its operational definition  (see tables 2 and 3).  

***Comment 3*** *Data related statistical methods that could be used in living reviews*

*There is also some literature about the statistical methods that could be used in living reviews (see Simmonds 2017). The Simmonds paper identifies many important methods that could be used. The authors have not indicated that they will collect data about different methods of statistical analyses and interpretation, but could. In addition, there is some question about the criteria to decide when re-analysis and a subsequent publication should occur when doing a living review (e.g., it could be when results or certainty of evidence change, or simply when there is new evidence regardless of whether the effect or certainty changes). Data about if and how the authors of the reviews report these methodological decisions would likely be important to collect.*

**Reply to comment 3** Even though the publication by Simmonds 2017 was included in our review of prior work done, we agree that still some variables related to statistical methods are needed. We have added new variables to collect specific information related to the statistical methods inspired in Simmonds 2017 work. *See: *“Data to be collected from the baseline report” in table 2- **Planning for evidence monitoring /surveillance, **and “Data to be collected from the updates” in table 2- **Information about the integration of new evidence (NEI)**)

***Comment 4*** *The methodological* *quality of the reviews are being assessed*

*It’s not clear to me why the methodological qualities of the reviews are being assessed. How will this information be used? In addition, do the current tools actually address issues relevant to living reviews? Will these tools provide quality information relevant to a living review in particular?*

**Reply to comment 4** A living systematic review should derive from a reliable systematic review, the "baseline review". Our aim in conducting the methodological quality assessment of both, the baseline report and their subsequent updates, is to assess if the methodological quality is maintained, improved, or decreased through its multiple updates. We agree with you, the AMSTAR II checklist, as well as the other instruments we have proposed to use, are not addressing the relevant issues of living evidence synthesis or reviews. Even though we will use them for this assessment because they do address important quality issues that any systematic evidence synthesis should meet. We have improved the explanation of this procedure in the text: Lines 217-225. To be able of identifying the methodological quality of the baseline reviews and any variations through its multiple updates, as part of the data collection we will assess the methodological quality of all included evidence synthesis reports using the appropriate instruments (i.e. AMSTAR II checklist for systematic reviews [21] and NCCMT Quality Assessment Tool-for other review articles [22]). Two independent authors will complete the assessment on the original publication (baseline review) and subsequent updated reports or publications. Disagreements will solve by consensus. We will pay particular attention to changes in the methodological quality through publications of the same review. Results of this assessment will be presented as part of the descriptive data in tables.

***Comment 5. Reviews’ nomenclature*** *A minor comment: I understand that different types of reviews will be included, but the paper may be easier to follow if the living 'reviews' are not referred to as ‘studies’.*

**Reply to comment 5** We have changed the references from “study/ies”  to “review/s” throughout  the manuscript. The change was introduced early in the text as follows: Line 154 Each report previously mentioned and the subsequent publications of the same review will be included and treated as a single study (i.e. review /evidence synthesis). After this, we use “review” for referring to the included evidence synthesis or reviews.

# Peer review: Reviewer response for version 1

**DOI:** 10.21956/openreseurope.15135.r27710

**Published:** 2021-10-06

**Authors:** Juan Erviti

**Affiliations:** 1 Unit of Innovation and Organization, Navarre Health Service, Pamplona, Spain

This is a really interesting project that can provide relevant added value in the realm of systematic reviews. The protocol draft is sound and well written. Please find below some suggestions to the authors:

***Search strategies***

 The draft reads as follows “ *An additional search using a highly sensitive search strategy will be performed on PubMed/MEDLINE, in order to compare results and be able to identify additional evidence synthesis reports not obtained from searches in Epistemonikos*”.

 This is intended to validate Epistemonikos as a database for searches on living reviews, including systematic reviews, network meta-analyses and living overviews. Since the main database for searching is Epistemonikos, it would be interesting to elaborate a little more about its quality and previous validation. Many readers may not be familiar to this database.

***Assessment of the methodological quality***

 Authors will evaluate the quality of all included studies (e.g. SRs, overviews, or network meta-analysis) using the AMSTAR II checklist and other appropriate instruments according to the type of study (e.g. the NCCMT Quality Assessment Tool-Review articles).

 Reference 21 in the draft is that of a previous version of the AMSTAR tool (published in 2007). Probably it would be better to swap the current reference 21 for the following:

 Shea B J, Reeves B C, Wells G, Thuku M, Hamel C, Moran J et al. AMSTAR 2: a critical appraisal tool for systematic reviews that include randomised or non-randomised studies of healthcare interventions, or both BMJ 2017; 358 :j4008 doi:10.1136/bmj.j4008

 An important limitation of the AMSTAR II tool is that the quality of individual studies in the meta-analysis is poorly evaluated. Domain 9 reads as follows: “ *Did the review authors use a satisfactory technique for assessing the risk of bias (RoB) in individual studies that were included in the review?*”

 There are two possible answers to this question, namely:

 For *Partial Yes*, must have assessed RoB from

- unconcealed allocation, and
- lack of blinding of patients and assessors when assessing outcomes (unnecessary for objective outcomes such as all cause mortality)

For *Yes*, must also have assessed RoB from:

- allocation sequence that was not truly random, and
- selection of the reported result from among multiple measurements or analyses of a specified outcome

Likewise, the NCCMT Quality Assessment Tool-Review provides a poor approach to the RoB assessment of individual studies in the review (described on Question 6 of the tool).

 Furthermore, in both AMSTAR II and NCCMT tools, the score in Domain 9 and Q6, respectively, is obtained simply if information on allocation, blinding, etc., has been *addressed*, regardless the resulting quality of individual studies included.

 This is really poor approach to the RoB assessment of individual studies included in the reviews which is a key aspect for the reliability of published results. At present, the Cochrane collaboration is piloting its RoB2 tool. Ideally, RoB in the individual studies of the reviews should be re-assessed with the help of better tools like RoB2. If not possible, this should be stated in the protocol as a limitation of the LE systematic review.

***Additional information on Table 2.***

 One of the variables included in Table 2 is the “Journal/Editorial Group/Publisher”. I would also include the “impact factor” in order to showcase the “visibility” of living evidence. Likewise, the “Journal/Editorial Group/Publisher” and “impact factor” of the last update could also be included in Table 3.

Is the study design appropriate for the research question?

Yes

Is the rationale for, and objectives of, the study clearly described?

Yes

Are sufficient details of the methods provided to allow replication by others?

Yes

Are the datasets clearly presented in a useable and accessible format?

Not applicable

Reviewer Expertise:

systematic reviews and pharmacoepidemiology

I confirm that I have read this submission and believe that I have an appropriate level of expertise to confirm that it is of an acceptable scientific standard, however I have significant reservations, as outlined above.

## References

[1] : Methods for living evidence synthesis: a systematic review protocol. *Open Research Europe* .2021;1: 10.12688/openreseurope.14044.1 10.12688/openreseurope.14044.1

[2] : AMSTAR 2: a critical appraisal tool for systematic reviews that include randomised or non-randomised studies of healthcare interventions, or both. *BMJ* .2017; 10.1136/bmj.j4008 10.1136/bmj.j4008 PMC583336528935701

# Response

**Published:** 2022-02-22

**Authors:** Maria Ximena Rojas Reyes

**Affiliations:** INSTITUT DE RECERCA DE L'HOSPITAL DE LA SANTA CREU I SANT PAU FUNDACION - IR-HSCSP, Spain

Dr. Erviti, we thank your careful reading of the manuscript and your helpful comments and suggestions. Please find below our reply to your comments and suggestions. (Reviewer comments in italics)

***Comment 1*** *Search strategies* *The draft reads as follows* “ *An additional search using a highly sensitive search strategy will be performed on PubMed/MEDLINE, in order to compare results and be able to identify additional evidence synthesis reports not obtained from searches in Epistemonikos*”.

*This is intended to validate Epistemonikos as a database for searches on living reviews, including systematic reviews, network meta-analyses, and living overviews. Since the main database for searching is Epistemonikos, it would be interesting to elaborate a little more about its quality and previous validation. Many readers may not be familiar with this database.*

**Reply to comment 1** We have decided not to carried out additional searches because Epistemonikos has been already validated for identification of any type of evidence syntheses. We have changed the paragraph and added a sentence with specific reference (reference  17). Lines 161-168: With this aim we will use Epistemonikos database as the main source; this is a comprehensive database of systematic reviews and other types of evidence synthesis reports, maintained by screening multiple information sources to identify SRs and their included primary studies, including the Cochrane Database of Systematic Reviews, Pubmed/MEDLINE, EMBASE, CINAHL, PsycINFO, LILACS, DARE, HTA Database, Campbell database, JBI Database of Systematic Reviews and Implementation Reports, EPPI-Centre Evidence Library [16]. This database has been validated, showing its completeness in collecting the published SRs and other type of evidence syntheses [17]*. *Reference 17: Rada, G., Pérez, D., Araya-Quintanilla, F. et al. Epistemonikos: a comprehensive database of systematic reviews for health decision-making. BMC Med Res Methodol 20, 286 (2020). https://doi.org/10.1186/s12874-020-01157-x  

***Comment 2*** *Assessment of the methodological quality* *Authors will evaluate the quality of all included studies (e.g. SRs, overviews, or network meta-analysis) using the AMSTAR II checklist and other appropriate instruments according to the type of study (e.g. the NCCMT Quality Assessment Tool-Review articles). Reference 21 in the draft is that of a previous version of the AMSTAR tool (published in 2007). Probably it would be better to swap the current reference 21 for the following: (1) Shea B J, Reeves B C, Wells G, Thuku M, Hamel C, Moran J et al. AMSTAR 2: a critical appraisal tool for systematic reviews that include randomized or non-randomised studies of healthcare interventions, or both BMJ 2017; 358 :j4008 doi:10.1136/bmj.j4008*

**Reply to comment 2** We have updated the reference in the protocol according to your suggestion: Reference 22. Shea B J, Reeves B C, Wells G, Thuku M, Hamel C, Moran J et al. AMSTAR 2: a critical appraisal tool for systematic reviews that include randomised or non-randomised studies of healthcare interventions, or both BMJ 2017; 358 :j4008 doi:10.1136/bmj.j4008

***Comment 3*** *Assessment of the methodological quality An important limitation of the AMSTAR II tool is that the quality of individual studies in the meta-analysis is poorly evaluated. Domain 9 reads as follows: “Did the review authors use a satisfactory technique for assessing the risk of bias (RoB) in individual studies that were included in the review?”* *There are two possible answers to this question, namely:*

*For Partial Yes, must have assessed RoB from*

- *unconcealed allocation, and*
- *lack of blinding of patients and assessors when assessing outcomes (unnecessary for objective outcomes such as all-cause mortality)*

*For Yes, must also have assessed RoB from:*

- *allocation sequence that was not truly random, and*
- *selection of the reported result from among multiple measurements or analyses of a specified outcome*

*Likewise, the NCCMT Quality Assessment Tool-Review provides a poor approach to the RoB assessment of individual studies in the review (described on Question 6 of the tool).*

*Furthermore, in both AMSTAR II and NCCMT tools, the score in Domain 9 and Q6, respectively, is obtained simply if information on allocation, blinding, etc., has been addressed, regardless the resulting quality of individual studies included. This is really poor approach to the RoB assessment of individual studies included in the reviews which is a key aspect for the reliability of published results. At present, the Cochrane collaboration is piloting its RoB2 tool. Ideally, RoB in the individual studies of the reviews should be re-assessed with the help of better tools like RoB2. If not possible, this should be stated in the protocol as a limitation of the LE systematic review.*

**Reply to comment 3** The goal of our analysis is to evaluate the methodological quality of the included SRs, from both the baseline one and their subsequent publications, but not the validity of the conclusions. We will then assess quality over time through the subsequent updates.

***Comment 4.*** *Additional information on Table 2. One of the variables included in Table 2 is the “Journal/Editorial Group/Publisher”. I would also include the “impact factor” in order to showcase the “visibility” of living evidence. Likewise, the “Journal/Editorial Group/Publisher” and “impact factor” of the last update could also be included in Table 3.*

**Reply to comment 4** We have added the additional information regarding the “impact factor” on journal publication Living evidence products in table 2. We believe this variable will help to summarize the scope of the journal and add value to our descriptive analysis.
